# Embrace the fat when getting old

**DOI:** 10.18632/aging.102341

**Published:** 2019-10-23

**Authors:** Lorenz Adlung, Ido Amit, Eran Elinav

**Affiliations:** 1 Immunology Department, Weizmann Institute of Science, Rehovot, Israel; 2 Division of Cancer-Microbiome Research, DKFZ, Heidelberg, Germany

**Keywords:** obesity, adipose, cardiometabolic disease, Trem2, macrophages

Aging is a complex and dynamic disease characterized by a gradual loss of physiological homeostasis, from the cellular to the organismic level, with ultimately fatal consequences. A more systemic understanding of the cell types and pathways at play is required for rational, targeted interventions into this progressing process [1]. Aging is a normal part of human life-span on the one hand, but associated with many disease risks on the other, indeed, the deterioration frequently exhibited during aging is considered a major risk factor for many human pathologies including type II diabetes mellitus and obesity. Immuno-metabolic derailments accompany the aging process and the development of obesity. Both phenomena therefore share many biological similarities including inflammatory insults and multi-layered cellular and subcellular aberrations [2]. Examples include altered intercellular communication, mitochondrial dysfunction and deregulated nutrient sensing; all hallmarks of aging that are also characteristic in the context of obesity. Among the other hallmarks of aging are stem cell exhaustion, loss of proteostasis, and cellular senescence [3]. The clearance of accumulating damaged cells or senescent cells through phagocytosis is a common strategy to counteract these harmful effects, including in the context of the aging process. As such, the immune system has been recently implicated as a central mediator of organ-level homeostasis and can thus help to preserve tissue integrity in anti-aging interventions. However, a fine balance is required to avoid uncontrolled over-activation of such beneficial immune responses, which may predispose to auto-immune consequences. The precise molecular-level characteristics of immune-metabolic interactions, including the involvement of subsets of phagocytes, and ways to specifically recruit and activate them to counteract aging-related metabolic risks and sustain homeostasis, still remain to be identified.

Recently, we have discovered a novel subpopulation of lipid-associated macrophage (LAM) cells [4]. By means of massively parallel single-cell RNA-sequencing and computational modelling, we generated a dynamic atlas of murine adipose tissue immune cells during the gradual transition from the lean to the obese state in mice fed a high-fat diet. We observed dominant changes occurring in the myeloid compartment, and identified Trem2, a triggering receptor expressed on myeloid cells, as a major, previously unrecognized driver of adipose-tissue immune-cell remodeling. Mainly originating from circulating monocytes, LAM cells arise in adipose tissue at the onset of obesity. They surround hypertrophic adipocytes in “crown-like” structures and express a conserved genetic program downstream of Trem2, which involves phagocytic processes such as lipid sensing, uptake and processing, as well as energy metabolism.

The core of the Trem2 genetic program is highly conserved as we found it also to be present in obese human adipose-tissue macrophages. Moreover, we detected LAM cells in the adipose tissue of a diet-independent mouse model of diabetes and obesity (*db*/*db*) as well as in the livers of wild-type mice on a high-fat diet. Interestingly, Trem2-positive macrophages were also described in the brains of mice and humans with neurodegenerative disease [5], and in the context of murine atherosclerosis [6].

Mechanistically, we found that the genetic ablation of Trem2 prevented the downstream activation of the Trem2 genetic program and thus LAM cell maturation and function in adipose tissue [4]. As a consequence, *Trem2-*depleted mice abrogated the recruitment of macrophages to be positioned around adipocytes. The deletion of Trem2 caused an enlargement of adipocytes, systemically elevated cholesterol levels, glucose intolerance and the loss of anti-inflammatory molecules. We concluded from these observations that LAM cells may play a prominent role in homeostatic adipose-tissue maintenance. Most likely, the impact of the Trem2 program extends beyond fat depots towards systemic properties of metabolic disorders, which might relate particularly to aging.

How could the Trem2 program be putatively harnessed to decelerate the aging process? The capability of LAM cells to prevent lipids from reaching the blood stream can be linked to aging, because systemic alterations in the lipid composition of mitochondrial membranes might perturb energy metabolism and through mitophagy also proteostasis and cellular turnover [7]. Furthermore, blocking the release of pro-inflammatory molecules from adipocytes in the presence of LAM cells may ameliorate the cellular deterioration during the aging process, including the associated inflammatory aspect, termed “inflammaging”. Many strategies in the aging field focus either on the clearance of damaged cells or senescent cells, or on the establishment of anti-inflammatory treatments [3]. There is a fine physiological balance between immune activation in the context of wound healing, infection and tissue development, and immune suppression to impede chronic inflammation and autoimmunity. This double-edged sword is also represented by the triggering receptor expressed on myeloid cells: While Trem2 exhibits anti-inflammatory properties, Trem1 was reported to promote inflammation. The pharmacological inhibition of Trem1 stimulated the expression of Trem2 on myeloid cells recruited to the ischemic mouse brain, thereby improving stroke outcome [8]. Future research will need to address whether the negative regulation between Trem1 and Trem2 is mutual. Besides the transcriptome, other regulatory and functional layers, such as the epigenome and the proteome need to be investigated. The complex nature of the aging process requires the scrutinization of non-immune cells and different layers at the organismic level; from the diet to processed, circulating metabolites.

**Figure 1:**
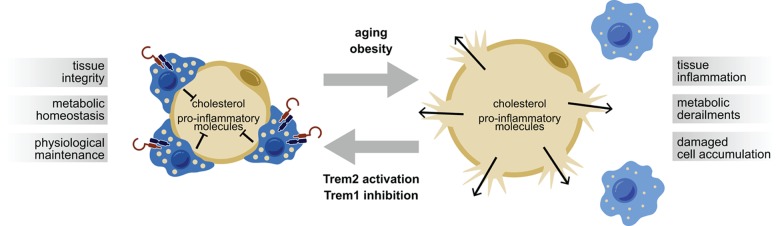
Lipid-Associated Macrophage (LAM) cells expressing Trem2 surround adipocytes in “crown-like structures” thereby preventing leakage of potentially harmful substances to the periphery with systemic consequences. Aging and/or obesity may eventually lead to a loss of LAM cells and thus bursting of hypertrophic adipocytes. Metabolic derailments could putatively be counteracted by activation of Trem2 or pharmacological inhibition of Trem1.

Taking this into consideration, we speculate that a LAM cell-based therapy might have an impact on aging-related disease and the aging process itself (Figure 1). In crown-like structures surrounding adipocytes, LAM cells prevent lipids and potentially pro-inflammatory molecules from reaching the periphery and causing systemic damage during aging. Thus “embracing the fat” will help to maintain tissue homeostasis when getting old.
